# Recovery of Water-Soluble Compounds from *Tisochrysis lutea*

**DOI:** 10.3390/membranes12080766

**Published:** 2022-08-05

**Authors:** Robin Lina, Olivier Lepine, Pascal Jaouen, Anthony Masse

**Affiliations:** 1 AlgoSource, 7 rue Eugene Cornet, F-44600 Saint-Nazaire, France; 2 Nantes Université, Oniris, Centre National de la Recherche Scientifique, GEPEA, UMR 6144, F-44600 Saint-Nazaire, France

**Keywords:** microalgae, membrane filtration, techno-economic, cosmetic, extracts

## Abstract

This work aims at studying the techno-economic feasibility to produce an extract, at a small industrial-production scale, from a *Tisochrysis lutea*’s paste, in view of cosmetic applications. The paste was first thawed, diluted and centrifuged to get a crude water extract. Then, two successive stages of membrane filtration were carried out: the first one to essentially remove/retain the particles (cellular debris) by microfiltration and the second one to concentrate (ultrafiltration) the soluble compounds of the permeate from the previous step. The robustness of the processing chain has been demonstrated following the production of three similar extracts with more than 30 L input material each. Around 54% of the final extract was composed of proteins and carbohydrates. The final ingredient was assessed for genomic activity and showed multiple positive responses. Finally, an economic analysis was performed, which demonstrated that the major cost is linked to centrifugation step. The total manpower represents the highest cost of the OPEX categories.

## 1. Introduction

Numerous microalgae and cyanobacteria are already marketed and industrialized for food, feed, energy, etc. [1,2]. They can constitute a source of proteins, carbohydrates, DocosaHexaenoic Acid (DHA), EicosaPentaenoic Acid (EPA) and natural compounds and also could replace some conventional chemical and synthetic compounds. The whole biomass can be marketed after growing, but their processing often offers more added value [3]. Specific proteins and sugars found in algae have already been extracted and valorised in previous papers [4]. Few works deal with their use for cosmetic use, their effect on the cutaneous dermis or their use for anti-aging prevention. 

Moreover, technical performances of extraction and recovery of microalga’s valuable molecules are often well-described in numerous publications, but just a few of them mention the economic aspects, in particular at high Technological Readiness Levels (TRL) whereas it is a crucial part of product development at industrial scale [5,6,7,8]. In fact, operations must be technically feasible as well as economically profitable. 

Microalgae strain *Tisochrysis lutea* is a small, brown seawater microalga belonging to the *Isochrysidacae* family with the most-known being *Isochrysis galbana* (IG) [9]. Despite a similar appearance, they are genetically different [10]. Both produce soluble proteins and carbohydrates as well as docosahexaenoic acid (DHA) and fucoxanthin; only *Tisochrysis lutea* shows no production of eicosapentaenoic acid (EPA) [11]. *Tisochrysis lutea* or *Isochrysis galbana* are already commonly used in aquaculture, but it could be interesting to study the feasibility of extracting soluble compounds as functional proteins and carbohydrates [12], since this algae specie does not have any cell walls and is easily disrupted [13]. 

Membrane filtration is often used to separate and concentrate various biomolecules, notably at an industrial scale, such as casein separation and purification. Membrane filtration allows operating with large volumes of solutions, while preserving the functionalities and nutritional properties of the molecules thanks to the mild operating conditions [14] and lack of added chemicals [15]. In the microalgae field, membrane technologies can be used to harvest microalgae [16] as well as to recover high-value compounds. Thus, for the previous cases, membrane filtration was investigated to isolate proteins from *Chlorella* [7,14] and to isolate and separate B-phycoerytrhin from carbohydrates [17,18,19]. As it was already experimented on Spirulina [20,21], both microfiltration and ultrafiltration can be used to isolate and concentrate soluble microalga’s molecules. Gerardo et al. [22] carried out a review that highlights the achievements, potential and challenges of integrating membrane technology into microalgae-based biorefineries.

In this paper, soluble proteins and carbohydrates from *Tisochrysis lutea* will be isolated, clarified and concentrated in order to produce one singular objectivated ingredient for cosmetic application. Experiments, repeated three times at TRL5 with volumes around 50 L, will allow to perform essential developments and analysis to consider further productions of an active extract at industrial scale. We, hereby, bring novelty summarising in one paper the robustness (reproducibility) of the developed process chain, the filtration reproducibility at large scale, the industrial scale productions results, the technical economic analysis and the final product activity.

## 2. Materials and Methods

### 2.1. Extraction and Recovery Methods

**Figure 1:**
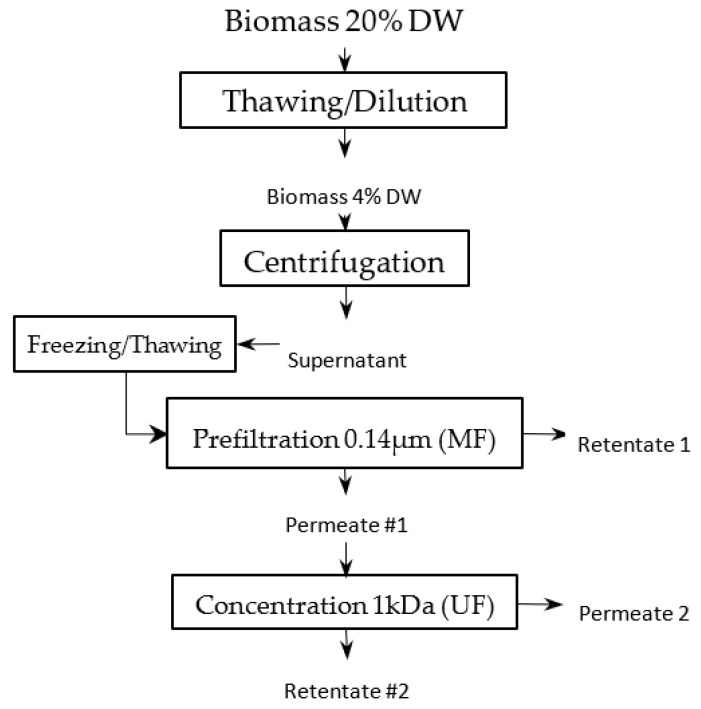
Filtration-process pathway.

**Figure 2:**
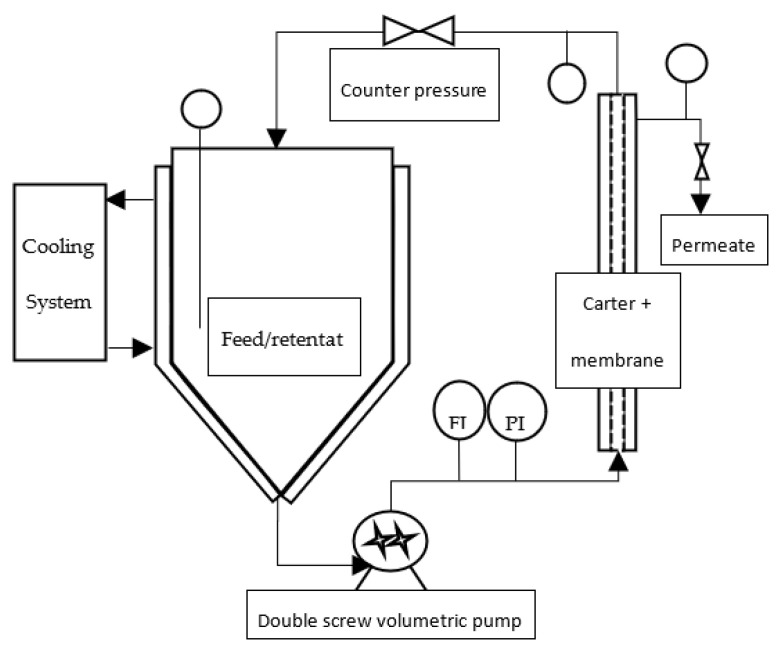
Filtration pilot design (FI: flow indicator; PI: pressure indicator).

One kilogram of a frozen paste of wild-type *Tisochrysis lutea* (supplied by Necton, Belamandil, Portugal) was thawed, then diluted 5 times with filtered tap water (0.2 μm pore size filter), in order to extract water-soluble material with reasonable concentration (~15 g·L^−1^) without adding supplemental salts that would reduce the purity of targeted molecules and increase the duration of the ultrafiltration step with several diafiltrations. A suspension with 40 g·L^−1^ dry matter was obtained. Then, this suspension was batch-centrifuged with 7–8 min residence time at temperature below 20 °C (GEA KG2006) at 11,000 g. The high-concentrated pellet (20% dry weight) was freeze-dried, while supernatant was stored frozen (−20 °C) to be further processed by filtration. This supernatant constitutes the raw material to be filtered (Figure 1). Each membrane filtration was carried out at 20 °C, with the supernatant thawed just before experiments (Figure 2). First, a microfiltration was performed at 1.6 bar with a 0.14 μm pore size ceramic membrane (23 channels, 0.35 m^2^, TAMI Industries MTB252311M014), in order to remove the remaining cell’s debris in the supernatant. At the end of the microfiltration, one diavolume of demineralized water was added (approximately 2.5 L) to extract more water-soluble compounds from the retentate. A previous experimentation, in which volume reduction and 3 consecutive diafiltrations (diavolume = dead volume) were performed, showed that 80% of the soluble-water compounds were extracted in the 2 first volumes. Since then, only 1 diafiltration was added. Then, the grouped permeates of the microfiltration step were submitted to ultrafiltration at 2.4 bar with a 1 kDa pore-size ceramic membrane (49 channels, 0.55 m², TAMI Industries MTB254911N001). Ultrafiltration was used in order to reduce the volume, to concentrate the soluble compounds such as large proteins and carbohydrates (>1 kDa, not measured) and to remove salts. During the filtrations, the mean cross-flow velocity within the membrane lumen was equal to 3 m·s^1^ due to double-screw pump equipment (Wangen TWIN70-43-1) limitation, and turbulent flow regimes were maintained. The experiments of filtration (micro- and ultrafiltration) were repeated 3 times in order to evaluate the reproducibility of operations. After each experiment of filtration, membranes were cleaned with the following protocol: recirculation of an alkaline solution, DEPTAL UF14 1%, during 45 min at 70 °C, recirculation of tap water at 70 °C with permeate valve closed, then recirculation of tap water at 70 °C with permeate valve opened until pH 7 reached (pH paper). New experiments were only carried out if the membrane-water permeability was 95% recovered after current experiment (~600 and ~15 L·h^−1^·m^−2^·b for microfiltration and ultrafiltration, respectively).

The permeate flux was calculated during each filtration with Equation (1):

J = V/Δt × A (1)

where V is the permeate volume in L obtained during Δt in hour, and A is the specific filtration area in m². J is expressed as L·h^−1^·m^−2^.

The Volume Reduction Factor (VRF) was calculated with Equation (2):

VRF = V_0_/(V_r,t_) = V_0_/(V_0_ − V_p,t_) (2)

In this equation, V_0_ is the initial volume to be filtered, and V_r,t_ is the volume of retentate at t time. The retentate volume can be calculated by using the cumulated permeate volume (V_p,t_) obtained at time t.

The retentate concentration was modelled following Equation (3):

C_R_ = C_0_ × (VRF)^RR^ (3)

where C_R_ is the concentration into the retentate tank (g·L^−1^), C_0_ is the concentration at the beginning of the filtration (g·L^−1^), and RR is the mean retention rate (%).

Retention Rate (RR) is defined as:

RR = 1 − C_p_/C_r_ (4)

C_P_ and C_R_ are the concentrations (g·L^−1^) at time t, respectively, in the instantaneous permeate and retentate.

The recovery rate of a compound has been calculated as follows:

Y_(X;Y)_ = (C_(X;Y)_ × V_(X;Y)_)/(C_0_ × V_0_) (5)

In this equation, X is either proteins or carbohydrates, and Y is either permeate (p) or retentate (r). C is the concentration of the molecules (g·L^−1^), and V is the volume in L. C_0_ and V_0_ are, respectively, the concentration (g·L^−1^) and the volume (L) in retentate at the beginning of filtration.

The Trans Membrane Pressure (TMP) was calculated with the following equation:

TMP = (P_e_ + P_s_)/2 − P_p_ (6)

P_e_ (bar) and P_s_ (bar) represent the retentate pressures, respectively, at the inlet and outlet of the membrane lumen, while P_p_ (bar) is the pressure on permeate side.

The purity (P_x_) was defined as the mass ratio between the targeted compound (m_x_) and the dry weight (m_dx_) of the sample:

P_x_ = m_x_/m_dw_ (7)

### 2.2. Analyses

Total proteins were analysed by the adapted Lowry’s colorimetric method (Lowry, 1951). Bovin Serum Albumin (BSA) was used as standard. Briefly, copper was reduced by proteins and bicinchoninic acid (Thermofisher BCA Assay kit, BCA1-1KT). The samples were analysed by microplate spectrophotometer (LX from Biotek, Winooski, VT, USA). Every sample analysis was performed in triplicate. Total carbohydrates were analysed by Dubois’s method (Dubois et al., 1956). This method includes sulphuric acid reaction with sugar and phenol addition to allow colorimetric quantification. D-Glucose was used as standard. Every sample analysis was performed in duplicate. The mineral (ashes) part of samples was determined after drying at 90 °C during 24 h and burning at 600 °C during 6 h. Every sample analysis was performed in duplicate. The particle sizes were measured with a Malvern Mastersizer 3000 granulometer. During genomic analysis, cells were plated at the concentration of 10,000 cells by well in 96-wells plates and incubated overnight, after which they were treated with the tested product for 24 h. We used qPCR microfluidic technology, in accordance with the protocol of Fluidigm. The chip we designed integrates genes that cover the entire range of skin activity. The RNAs were extracted using the mRNA CatcherPLUS Kit (Thermofisher, Waltham, MA, USA). Afterwards, total mRNA was reverse-transcribed to cDNA using the High-Capacity cDNA Reverse Transcription Kit (ThermoFisher, Waltham, MA, USA). A preamplification step was carried out with all the primers used in the chip and the cDNA that has been deposited on the chip. The mix blending was undertaken by the IFC Controller. The chip was placed in the BioMark, and qPCR amplifications were performed using TaqMan gene expression master mix/EvaGreen system (ThermoFisher, Waltham, MA, USA) in accordance with the protocol of the manufacturer.

## 3. Results

### 3.1. Microfiltration Experiments

**Figure 3:**
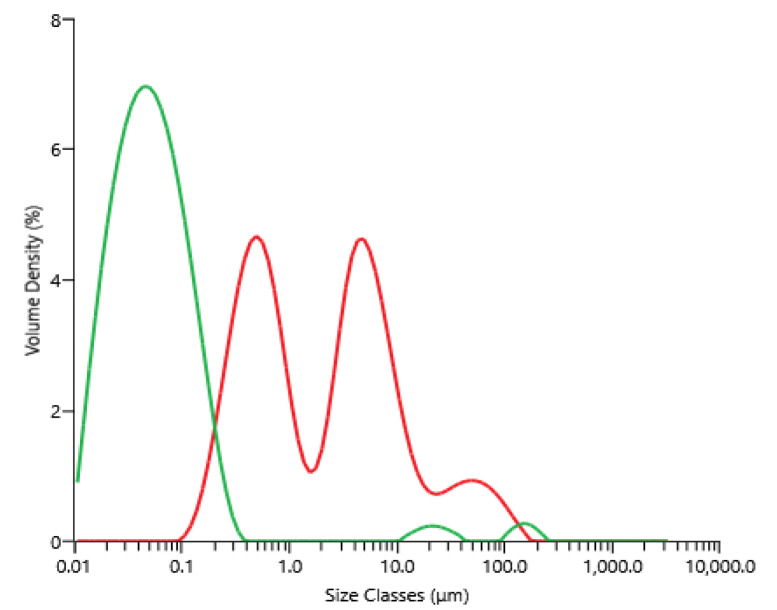
Volume percentage as a function of particle size for the supernatant obtained after centrifugation and freezing/thawing equation before microfiltration (red), and the permeate obtained after microfiltration (green).

The supernatant of centrifugation (raw material for microfiltration) contained between 3.0 and 3.8 g·L^−1^, 2.1 and 3.0 g·L^−1^, 5.0 and 5.5 g·L^−1^ and 14 and 17 g·L^−1^ of proteins, carbohydrates, ashes and dry matter, respectively; these concentrations depended on the microalga’s paste lot, which was thawed and centrifuged. Figure 3 shows the volume percentage as a function of the particle diameter present within the supernatant obtained after centrifugation, then freezing and thawing, as well as the permeate obtained after microfiltration. The microfiltration allowed a clarification of the supernatant, since particles bigger than 1 µm found initially in the feed were not present in the product, the permeate. For information, contrary to the brown and opaque supernatant of centrifugation, the permeate of the microfiltration appeared clear and yellow-orange.

**Figure 4:**
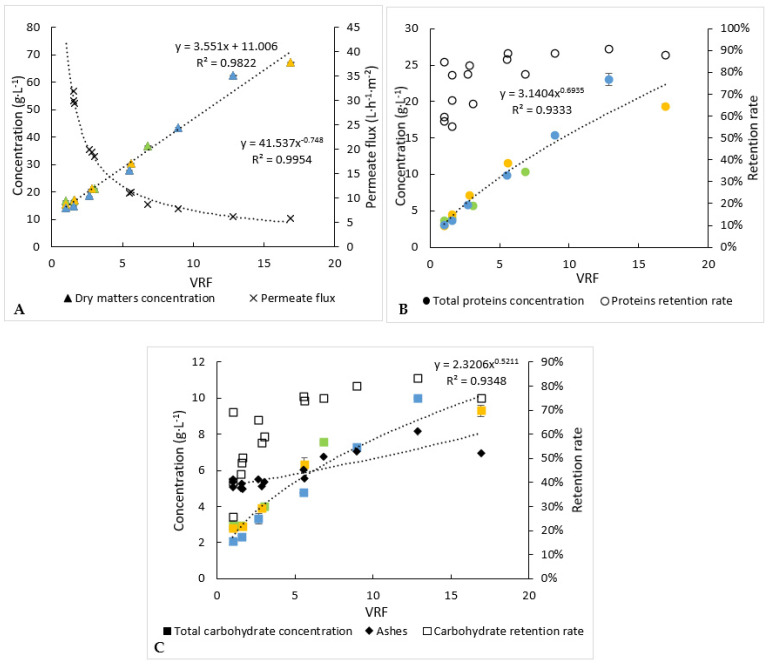
Biochemical follow-up of retentate during the 3 microfiltration experiments (each experiment is represented by one colour). (**A**) Dry-matter concentration and permeate flux, (**B**) analysis of proteins and (**C**) analysis of carbohydrates. Error bars correspond to the standard variations between 3 replicates for proteins and 2 replicates for carbohydrates and dry matter.

The microfiltration allowed to reach a high-volume reduction factor (VRF) equal to 17 after only 8.4 h of continuous operation (Figure 4A). During this period, the mean permeate flux was equal to 16 L·h^−1^·m^−2^ (Figure 4A). Filtering a 50 L batch of solution several times could be considered as a TRL5 range of experiments. A full day of 7 h filtration with a 10 m^2^ (approx. 30 membranes) filtration area could allow the processing of 1120 L of solution. Additional developments (in scale), optimisations (automatic backwash after a low permeate flow was reached) and cost analysis could lead to the industrial production of high-value molecules. 

Concentrations of proteins, carbohydrates and dry matter into the retentates (Figure 4) and permeates for the 3 microfiltration batches have been determined over time. 

The protein and carbohydrate concentrations in the retentates increased from 3.1 g·L^−1^ and 2.3 g·L^−1^ to 22.4 g·L^−1^ and 10.2 g·L^−1^, respectively, at the end of operation. The permeate concentrations of proteins, carbohydrates and dry matter remained relatively constant (results not shown), while they increased into the retentates. The mean retention rate deduced from equation (3) of the carbohydrates (52%) was lower than that of the proteins (69%). Consequently, the concentration factor of the carbohydrates was the lowest, around 4.4. It is worth noting that a part of the proteins or carbohydrates could be attached to the cellular debris, which would prevent their passage through the membrane, and retention rates variations between VRF 1 to 4 may be explained by the slight variations on molecule concentrations in the starting solution and a “maximal” and constant concentration of molecules reached in the permeate. Moreover, the retention rates increased for all compounds as the volume of retentate was reduced (Figure 4). This could be due to membrane fouling and/or a change into the polarisation layer at the membrane surface.

In an interesting and desired way, dry-matter concentration into the retentate increased the fastest, comparatively, to the protein and carbohydrate ones. Thus, compounds other than proteins and carbohydrates would be preferentially retained. The slight increase in ash concentration within the retentates could mean that cell debris would contain minerals. 

Each experiment of microfiltration has not been stopped at the same value of the volume-reduction factor (Figure 4). In addition, slight changes of compositions of the initial microalga’s paste were observed between the three experiments of microfiltration. Consequently, to level out these variations and propose a significant mass balance in line with the average behaviour of microfiltration, the evolution of the concentrations of compounds was modelled following equation 3. From these models, the final concentrations within the retentates, at the volume reduction factor equal to 17 (case of one experiment), were calculated (Table 1). For proteins and carbohydrates, they were equal to 22.4 and 10.2 g·L^−1^, respectively, while their concentrations within the permeate were rather stable during the microfiltration and equal to 1.3 g·L^−1^ (±0.09) and 1.5 g·L^−1^ (±0.01), respectively (Table 1). Thus, it appears that, at least, 35% of proteins did not pass through the membrane versus 20% of carbohydrates. The retentate obtained at the end of the microfiltration should be subsequently upgraded/treated, due to the potentially interesting remaining molecules.

Even if the ashes concentration in the retentate increased smoothly for reasons given earlier in this paper, 78% (Table 1) was recovered in the bigger volume, the permeate. It should be noted that all the material was not recovered within the retentate and the permeate, since the sum of the permeate and retentate recovery rates were not equal to 100% (Table 1). Thus, approximately 33% of proteins and carbohydrates seem to remain in the experimental setup. A part could participate to the fouling of the membrane. 

### 3.2. Ultrafiltration Experiments

**Figure 5:**
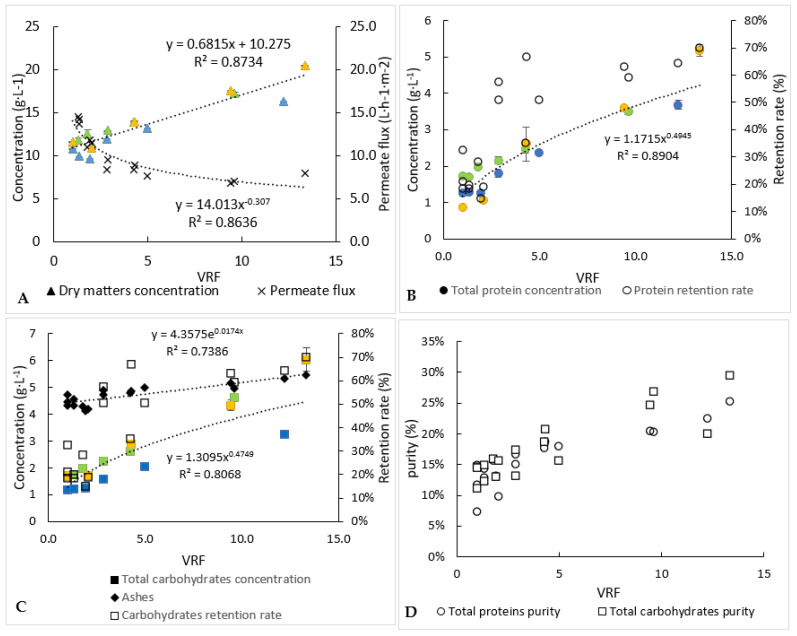
Biochemical follow-up of permeate and retentate during the 3 ultrafiltration experiments (each experiment is represented by one colour). (**A**) Dry matter concentration and permeate flux, (**B**) analysis of proteins, (**C**) analysis of carbohydrates and (**D**) index of purities. Error bars correspond to the standard variations between 3 replicates for proteins and 2 replicates for carbohydrates and dry matter.

All the permeates obtained with the three previous microfiltration experiments were concentrated by ultrafiltration (Figure 5). It took 8.7 h to reduce the retentate volume by 13 (Table 2). Compared to the microfiltration step, the same permeate flux evolution was observed during the ultrafiltration step. At the end of ultrafiltration, the pseudo-stabilized permeate flux was equal to around 6 L·h^−1^·m^−2^. This value could be considered relatively low, but it should be noted that the ultrafiltration was carried out up to a high-volume reduction factor.

As indicated previously (Table 1), the operation of ultrafiltration started with a juice of around 1.3 and 1.5 g·L^−1^ of proteins and carbohydrates, respectively. The concentrations of the retentate increased as the volume decreased (Figure 5), to reach 4.3 and 4.4 g·L^−1^ for proteins and carbohydrates, respectively (Table 2). Thus, similar behaviour seems to occur for proteins and carbohydrates, which is why similar average retention rates were found for these compounds (47–49%). These retention rates were lower than those obtained during microfiltration (69% for proteins against 52% for carbohydrates), while a lower molecular-weight membrane cut-off threshold was used, but it should be remembered that during the first filtration, part of the proteins and carbohydrates was probably still attached to the debris of the cell versus being “free” in the case of the ultrafiltration step. Unfortunately, too many proteins and carbohydrates passed into the permeate of ultrafiltration, due to the duration and the temperature of filtration, so microbial development may have occurred (not measured) and resulted in degradation of large molecules into small ones, small enough to go through our 1 kDa ultrafiltration membrane; a detailed analysis of proteins before and after processing may answer the question. Thus, around 82% and 72% of the proteins and carbohydrates, respectively, were recovered into the whole of the permeate recovered at the end of the ultrafiltration. In order to avoid the loss of these compounds, it could be interesting to change the 1 kDa ultrafiltration membrane to a smaller pore-size membrane with a different material. Analysis (size, type and molecular weight) of the water-soluble compounds retained in the retentate and in the permeate would help to carefully select the best membrane. Similarly to the microfiltration experiment, most of the ashes (94%) were recovered in the permeate (Table 2). Not all of the dry matter was recovered in either the permeate or the retentate. A part of this matter could participate in the membrane fouling.

The overall process of separation and purification by microfiltration and ultrafiltration allowed to recover 10.6% and 14.7% of the proteins and carbohydrates, respectively, which were initially present in the centrifugation supernatant. The rest of these molecules can be found in the microfiltration retentate and in the ultrafiltration permeate.

Even if too many proteins and carbohydrates were lost, it should be noted that the ultrafiltration step in concentration mode (reduction of the volume of the retentate) made it possible to eliminate more “impurities” than the targeted compounds (proteins and carbohydrates). Consequently, the purity index increased from 7% to 25% and 12% to 29%, respectively, for proteins and carbohydrates.

Finally, it should be noted that the adopted methodology for the extraction and recovery of compounds allows to guarantee a certain reproducibility of operations. In fact, from the graphs of the Figure 4 and Figure 5, it appears that all the experimental points were close to the trendline; thus, all the correlation coefficients were higher than 0.8. With a view to market an ingredient, this makes it possible to guarantee a certain standardization of the composition of the ingredient. We, therefore, achieved our objective, developing a robust process that can be still optimised.

### 3.3. Bioactivity

A genomic analysis of a final retentate (after micro- and ultrafiltration) was performed by the French company Syntivia. This sample contained 3.5 g·L^−1^ of proteins, 3.3 g·L^−1^ of carbohydrates and 3.7 g·L^−1^ of ashes. The number of genes responding positively to the presence of the sample as a function of the effect and the type of cell are summarized (Table 3). The results showed various positive responses on dermal and epidermal cells and for different gene types (not disclosed). Dermal cells were the most receptive and exhibited 18 positive responses, especially for preventing anti-aging. On the overall genes tested to realise this, as shown in Table 3, positive responses represent 81% among all the genes tested.

Dermal cells exhibited the highest number of positive responses. Thus, given such a claim, after regulatory and toxicity control, this extract could provide to the market a new ingredient to produce cosmetics. Further tests and analysis may be performed to confirm and precise these effects before being used in formulations processes.

### 3.4. Economic Aspect

A financial evaluation, including CAPEX and OPEX costs, is proposed on the basis of the results of this work. Electricity, chemicals, water, workforce and raw material (microalga paste) were considered for the OPEX evaluation.

CAPEX is composed of all the equipment used for the entire process, which means thawing and dilution tanks, centrifuge, pumps, freezer and filtration pilot. Among the total CAPEX costs of the process, 1%, 62% and 38% are, respectively, represented by thawing, centrifugation and the filtration step, including microfiltration and ultrafiltration. Centrifuges are quite expensive equipment, so, in the project MAGNIFICENT, they were used to obtain a solid fraction from which lipids could be valorised. For a higher-production scale, filtration equipment for the supernatant could take the lead of all the CAPEX costs, due to the filtration surface and capacity raising.

**Figure 6:**
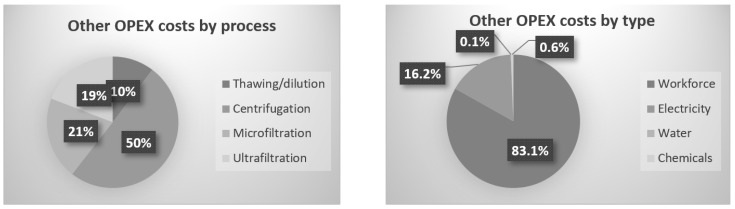
Repartition of OPEX costs as a function of the process unit and type.

For all steps, the OPEX costs were measured or calculated. It was, consequently, possible to simulate the overall production cost for a targeted amount of product and a targeted concentration of valuable molecules such as total proteins. Figure 6 represents the costs’ repartition for the experiment with a microfiltration VRF of 17 and an ultrafiltration VRF of 11.5. The obtained concentration in the product after ultrafiltration was 4.1 g·L^−1^. For the calculations, the workforce, electricity and water costs were established to be 20 EUR·h^−1^, 0.18 EUR·kWh^−1^ and 1.5 EUR·m^−3^, respectively. The electric cost was calculated as a function of the power and the time of use for each equipment (centrifuge, pump and cooling system). Water cost depends on the volume used for dilution and for installation cleaning. Workforce cost is established with the time spent on the process.

OPEX = ∑ (electricity + water + workforce + chemicals)

For the overall production cost, 85% is taken by the initial microalgae biomass; for that reason, all the extract losses have a heavy impact on the process and finally on the product cost. The last 15% is divided between the downstream processes and cost sources, as is presented in Figure 6.

The batch centrifuge represents the highest cost due to the time-consuming efforts between batches to collect the solid fractions. Instead of centrifuge, a first harvest by filtration could have been used; for project MAGNIFICENT, the pellet had to be concentrated enough (~20% dry weight) to avoid high volume for further freeze-drying and downstream processes. Supernatant membrane filtration, the main subject of this paper, is coming after, in terms of costs. Microfiltration and ultrafiltration combined represent 40% of the processes’ costs, without the biomass cost included. Like in publication [7], which consisted of filtration and diafiltration on chlorella, the workforce cost is the highest. In contrast, water and electricity are inverted in the costs ranks, which is explained by the higher scale, where electricity needs are higher. Moreover, water consumption is lower due to the absence of diafiltration. 

Within the conditions applied for this experiment, we could expect two filtration batches per week, corresponding to approx. 6 L of product. The annual production would be around 324 L of clarified and concentrated solution, containing up to 4–5 g·L^−1^ proteins and carbohydrates. At this state, the CAPEX is almost equal to the OPEX for 1 year of production. A bigger installation would raise the production capacity sharply. 

From a scaling-up perspective, we can link the final desired extract to the initial biomass need, considering all the parameters of the process. For ultrafiltration, efficiency is determined by the retention rate of the targeted molecule. The equation below calculates the VRF to be applied during ultrafiltration for a targeted extract concentration C, with RR, the retention rate of soluble compounds with an ultrafiltration membrane.

VRF=(C/C_0_)^1/RR^

Once the VRF is known, we can use the targeted extract volume and simple division to find the amount to be filtrated. The same calculations can be made for microfiltration. Once the amount to be filtrated with microfiltration is known, it is possible to throw back by mass balances to the initial biomass needs, including transformation by the previous processes. Any change in the previous process steps can impact this equation, and modification of the raw biomass-dilution rate would impact C_0_ for both filtration steps, the fouling rate and the permeate flowrate. For example, reducing the biomass-dilution rate would increase the concentration of the compounds and debris in C_0_ for microfiltration; the fouling would be faster, but the concentration of molecule in the permeate would be higher, and the processing time may be equal because of the reduction of the volume to be filtered. More experiments are needed to confirm these hypotheses.

Indirectly, the filtration efficiency has a very high impact on the overall product cost. Increasing the retention rate of the targeted molecule would prevent losses of it, but would also reduce the amounts to be filtrated and, thus, the upstream amounts, including the initial biomass, which is the highest cost of the process. Increasing the retention rate of the ultrafiltration by 20% can reduce the price per litre of product by 31%. The biomass fraction among the overall costs will also decrease due to some fixed costs from the process’ incompressible duration, cleaning, etc.

## 4. Conclusions

This paper’s subject concerned the clarification and the concentration of a liquid extract obtained from disruption by the freezing/thawing and centrifugation of *Tisochrysis lutea*. The first lab experiments allowed to evaluate the feasibility of this operation from two steps of membrane filtration. Microfiltration successfully removed cells’ particles and produced a clear orange permeate. This permeate was then successfully concentrated with an ultrafiltration at 1 kDa. Thus, the concentrations of proteins and carbohydrates have been increased three to five times. 

Microfiltration and ultrafiltration were conducted, in the end, three times. The biggest interests of this paper are the large-scale experimentations that allowed to obtain a Volume Reduction Factor (VRF) higher than 17 and 13, respectively, for microfiltration and ultrafiltration and the production of a clarified microalgae-based soluble ingredient. 

A bioactivity analysis showed some interesting responses for several genes in both the dermal and epidermal areas.

Such large-scale experiments are very convenient to gain an idea of the CAPEX and OPEX costs for potential industrial-scale production. In our case, for *Tisochrysis* *lutea*, some additional studies should be made to ensure a lower production cost. For treatment of the biomass such as pH modulation, cell disruption could first increase the release of a soluble molecule. Secondly, an effort regarding the filtration steps could help in using less material to produce the targeted amount of product or to acquire the desired concentration of product.

## Figures and Tables

**Table 1:** Mass balance carried out following the microfiltration step.

Initial Volume	50 L				
VRF max	17				
Jmean	16 L·h^−1^·m^−^²				
Filtration surface	0.35 m²				
Duration	8.4 h				
Concentrations (g·L^−1^)	Mean feedstock *	Final retentate *	Final permeate	Yr (%)	Yp (%)
Total proteins	3.1	22.4	1.3 ± 0.09	35	32
Total carbohydrates	2.3	10.2	1.5 ± 0.01	20	47
Total ashes	5.2	8.1	4.5 ± 0.01	9	78
Dry matter	14.6	71.4	11.3 ± 0.02	25	64

* The concentrations into the feedstock and the final retentate have been estimated from Equation (3).

**Table 2:** Mass balance carried out following the ultrafiltration step.

Initial Volume	50 L				
VRF max	13				
Jmean	10 L·h^−1^·m^−2^				
Filtration surface	0.55 m²				
Duration	8.39 h				
Concentrations (g·L^−1^)	Mean feedstock	Final retentate	Mean final permeate	Yr (%)	Yp (%)
Total proteins	1.2	4.3	1.07 ± 0.18	27	82
Total carbohydrates	1.3	4.4	1.02 ± 0.07	26	72
Total ashes	4.4	5.5	4.5 ± 0.16	10	94
Dry matter	10.9	19.1	9.6 ± 0.30	13	81

* The concentrations into the feedstock and the final retentate have been estimated with Equation (3).

**Table 3:** Genetic response in presence with clarified Tisochrysis and concentrated water extract.

	Curative Anti-Aging	Preventive Anti-Aging		Preventive and Curative Anti-Aging
Dermal cells	5	6	7	0
Epidermal cells	0	0	4	4

## Data Availability

Not applicable.
